# Towards Sustainable Food Security through Regional Grain Supply and Demand Analysis in China

**DOI:** 10.3390/ijerph20043434

**Published:** 2023-02-15

**Authors:** Tian Hu, Zhengshan Ju, Xiaoyang Liu

**Affiliations:** 1 Beijing Key Laboratory of Megaregions Sustainable Development Modelling, School of Urban Economics and Public Administration, Capital University of Economics and Business, Beijing 100070, China; 2 Technology Innovation Center of Land Engineering, Ministry of Natural Resources, Beijing 100035, China; 3 Technical Centre for Soil, Agriculture and Rural Ecology and Environment, Ministry of Ecology and Environment, Beijing 100083, China

**Keywords:** sustainability, food security, regional disparity, cities, cultivated land

## Abstract

As a vital part of sustainable development, food security is challenged by prolonged and concurrent pressures. Efforts have long been devoted to balance grain production across China as a whole, and thereby the uncertainties and underlying crisis in the regional grain-producing systems are hidden. In this study, we characterize the dynamic evolution of 357 cities and explore the dominant supply and demand effects to signal early warnings of grain insecurity. Our results show that 220 cities are in unsustainable grain supply–demand conditions in comparison with 10 years ago. Additionally, the south and southwest of China have experienced enlarged disparities and more severe grain insecurity. The dual effects from both increased population and decreased grain output are substantially responsible for the unsustainable grain-producing system on the city scale. Moreover, cities identified as having grain insecurity occupy high-quality cultivated land, including 55.4% of top-grade land, 49.8% of high-grade land, and only 28.9% of low-grade land. We consequently inform the incongruity between grain productivity and regional grain conditions. It is suggested that current intensive management of cultivation and the strategy of differentiated responsibilities in grain production should be based on environmental sustainability and a degree of self-sufficiency across the region.

## 1. Introduction

The grain security initiative is a global effort to end hunger and alleviate malnutrition. Concurrent risks, including climate change, the COVID-19 pandemic, and the Ukraine–Russia war, have triggered the vulnerability of the grain system and the instability of grain supply [1]. In response to such multi-layered crises, global grain security is reminded of the ability to access raw materials from the local to global scale [2].

It is challenging to characterize grain security given its many links and influencing factors. Worldwide, efforts have been made to depict grain security as aligning with concerns about how to feed the population [3]. The United Nations Food and Agricultural Organization declares grain security as having four pillars: availability, accessibility, utilization, and stability [4]. Such a broad concept is widely accepted but is challenged in practice to precisely describe what grain security is. Instead, grain insecurity could be defined as when conditions fall short of one or several facets [5]. It is commonly depicted as the large gap between supply and demand or low grain self-sufficiency [6,7]. In this sense, the grain system could be anchored by two endpoints of supply and demand. The cultivated land is emphasized in specific studies as more related to grain production capacity, thus advocating the characterization of the grain system from the supply side [8,9]. The alternative opinion supports the concept that grain insecurity is more about a consumption issue that is affected by the price of food, the dietary habitats, the population, etc. [10]. As projected in previous studies, the influences of the grain demand side on the grain system are looming large due to unprecedented urban sprawl and the aggregated population [11].

The grain security of China is important for feeding a considerable population, but it is suffering from the pressures of economic prosperity and sustainable agriculture. National grain supply and demand have long been in a delicate balance, underpinned by the principle of basic grain self-sufficiency. Aggregated research is based on analysis of grain self-sufficiency at the national level and lacks the regional variability and potential to increase sustainability [12]. Some problems arise afterwards when the lens is adjusted from the whole system to the regional scale. One is the change in grain output patterns. It has been demonstrated that the majority of grain production in China has been transferring from the south to the north since the 1990s [13]. Meanwhile, northern and northeast China have overtaken the Yangtze River Basin as the dominant cereal suppliers [14]. Another long-standing problem in the regional grain system is the severe imbalance of grain demand [15], which is somewhat induced by the intensified agriculture strategy associated with differentiated responsibilities in grain production. It has been asserted that populous regions have sufficient financial and workforce resources but assume limited responsibilities in grain supply. 

Given that grain supply–demand conditions have been associated with actual uncertainties and crises on a regional scale, we focus our attention on the evolution of the grain production system in the cities of China by integrating spatio-temporal variances, with the aim of inspiring long-term stability and sustainable development of the grain production system. The trends of grain supply–demand conditions in 357 prefecture-level cities were estimated from 2008 to 2018, and 220 cities were identified as having four types of grain insecurity. The determinants of unsustainable grain systems are explored further from the perspectives of population, grain output, and the distribution of cultivated land. Our findings are expected to illuminate the regional problems behind the perfect national balance between grain supply and demand. 

## 2. Materials and Methods

### 2.1. Data Sources

This study used data including the total permanent population (year-end), the total grain yield, and the graded cultivated land. The data for the population and grain yield from 2008 to 2018 were sourced from the National Bureau of Statistics of China. The data for cultivated land on a scale of 1:1,000,000 were sourced from the database provided by the Ministry of Natural Resources. The quality of cultivated land was estimated and classified into 15 levels according to the conditions of fertility and utilization. The cultivated lands with levels 1 to 4, 5 to 8, 9 to 12, and 13 to 15 are defined as the top grade, high grade, medium grade, and low grade, respectively. 

There are a total of 357 cities on the municipalities and prefectures scale. Several cities have been left out due to missing data, including Shihezi, Beitun, and the other seven cities in Xinjiang province, and Dongsha islands in Guangdong province. Additionally, 13 producing regions, 7 consuming regions, and 11 balanced regions on the provinces scale were identified in the mainland of China. Therein, the producing areas assume the primary responsibility for the domestic grain production as suppliers across the mainland of China. 

### 2.2. Methods

The general approach in this study included three parts. To begin, we evaluated the grain supply–demand conditions of 357 cities in 2008 and 2018 based on grain yield and permanent population. Additionally, the variations in grain supply–demand conditions from 2008 to 2018 were calculated. Furthermore, we identified 220 cities with grain insecurity. Lastly, the determinants for grain insecurity were explored from the supply and demand sides. 

Evaluate grain supply–demand conditions in 2008 and 2018. The condition of grain supply–demand per year is indicated by the gap between gross grain supply and grain demand. The grain supply was directly proxied by the grain yield, while the grain demand was calculated as the per capita share multiplied by the permanent population. We adopted the international standard line per capita share, which is advised as 400 kilograms for basic food security. Consequently, the grain supply–demand condition is described as two types: grain surplus and grain deficit. 

**Figure 1:**
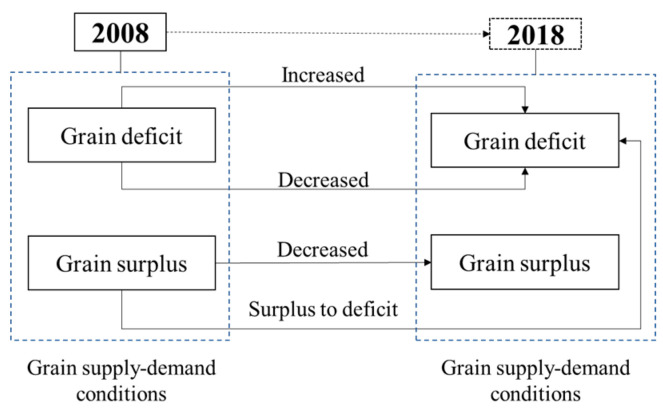
Four types of grain insecurity based on variations in grain supply-demand conditions from 2008 to 2018.

Identify grain insecurity based on changes in grain supply–demand conditions. Variations in grain supply–demand conditions were calculated from 2008 to 2018. There exists a total of four types of grain insecurity (Figure 1). When grain supply–demand conditions in 2008 and 2018 were in deficit, they were identified as grain insecurity, including the increased and decreased trends. Some cities presenting grain surpluses in 2008 and 2018 were identified as grain insecurity for the decreased trend. There were absolute changes in the grain supply–demand conditions from 2008 to 2018, indicating the unsustainability of the regional grain system. 

Diagnose the determinant of grain insecurity. The determinants of grain insecurity were revealed from the supply and demand sides. On the supply side, grain output and the distribution of cultivated land were taken into account. Variations in population were introduced for the demand side of the regional grain system. We describe the relationship between the population and grain output after calculating their standard scores. Normalized variations in population and grain output in 220 cities were obtained by the zero-mean normalization method. The standard value of population and grain output may be positive or negative, with a positive value indicating that the variation lies above the mean, and a negative value indicating that it lies below the mean. Additionally, the variations in population and grain output were compared to identify their contributions to the regional grain system. If the standard value of the population is larger than the grain production, cities could be featured as population-induced, and vice versa. On the supply side, we further aligned the quantity and quality of cultivated land with the types of grain insecurity to analyze the mismatch between grain productivity and grain supply–demand conditions.

## 3. Results

### 3.1. Enlarged Regional Disparities Based on Variations in Grain Supply–Demand Conditions 

**Figure 2:**
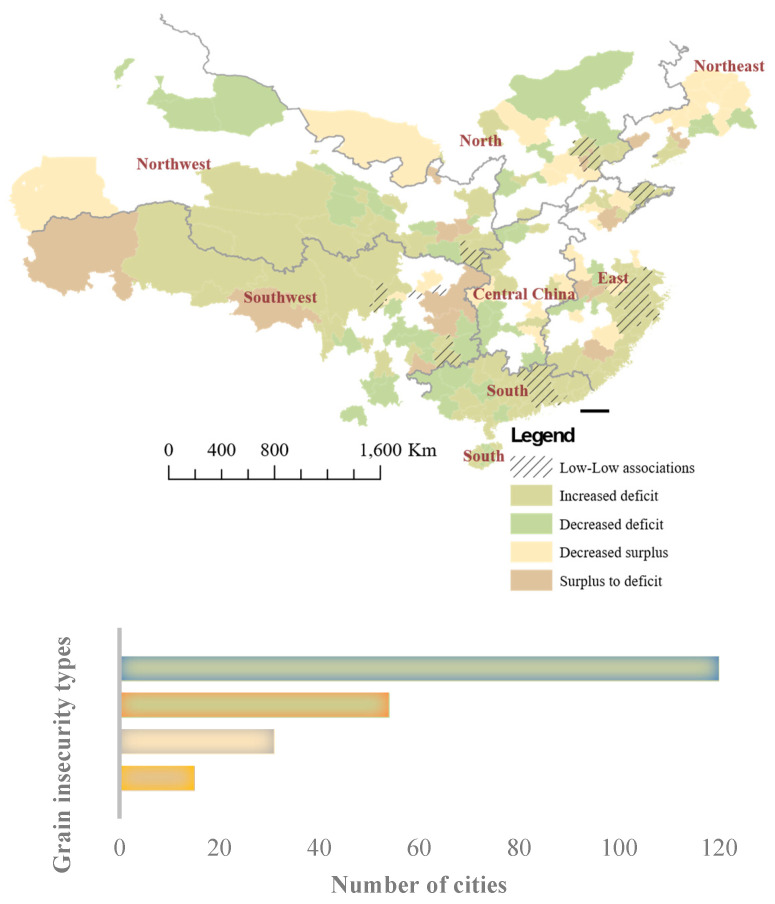
Distribution and number of cities with grain insecurity based on variations in grain supply–demand conditions from 2008 to 2018. Where the oblique line denotes the low–low associations, hotspots of grain insecurity are indicated. The total of 357 cities is disaggregated by grain supply–demand group.

A large share of cities was identified as having grain insecurity across China, which was larger than the amount of grain security (Figure 2). This shows that a total of 220 out of 357 cities were experiencing grain insecurity from 2008 to 2018. Thus, 120 cities saw an increase in grain deficit. Another 54 cities saw an opposite decrease in grain deficit, indicating a slight improvement in the regional grain supply–demand conditions. A total of 15 cities experienced absolute changes from surplus to deficit, indicating unsustainable grain supply–demand conditions. There were 31 cities that had grain surpluses in both 2008 and 2018, while they showed decreasing trends during those periods and hence were identified as having grain insecurity. The remaining 38.4% of the cities were portrayed as having grain security with increased surpluses and changes from deficits to surpluses. 

The regional scale of the disparities increased during the period, and the unsustainable conditions of grain supply and demand mainly were distributed in southern and southwest China. Specifically, the largest share of cities identified as experiencing an increase in grain deficit was in southwest China and coastal areas of the south and east. Cities with a decrease in grain deficit were scattered across a broad area of China. The decrease in grain surplus was specifically located in the boundary zones in central China. It was noted that 29 out of 31 cities with a decreased surplus were located in the main producing areas. For the changes from surplus to deficit, 5 of 15 cities were located in the southwest, and 6 were located in the main producing areas. The hotspots of low–low associations of grain insecurity were identified. These were predominated by an increased grain deficit, indicating the aggregation of grain insecurity. There were two cities in the east and north that were specially identified as having low–low associations. One was Xuancheng city in Anhui province, which had a decreased grain surplus as well as neighboring cities with grain insecurity. The other was Langfang city in Hebei province, which was a result of the change from surplus to deficit. 

### 3.2. Dual Effects from the Population and Grain Output on Regional Grain Insecurity 

**Figure 3:**
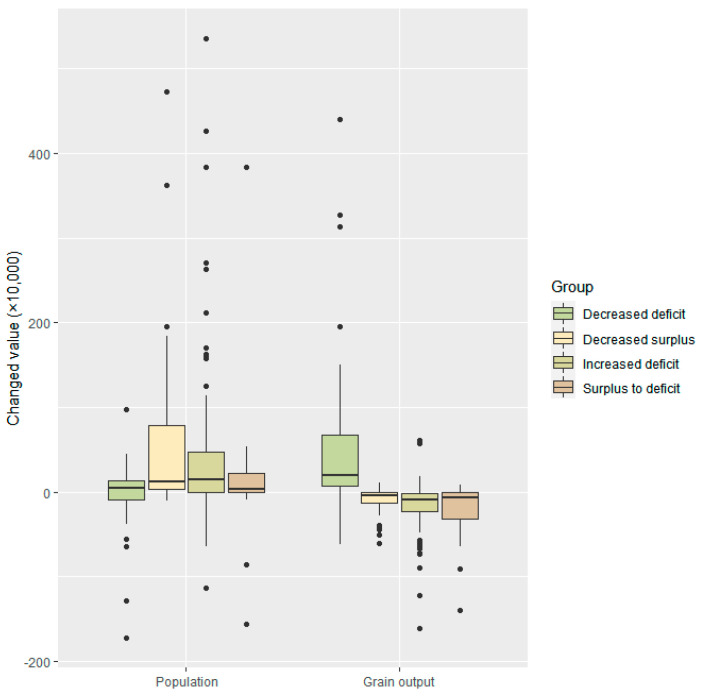
Variation of the total population and grain output. The vertical axis indicates the variation of the aggregate population and grain output from 2008 to 2018. In a boxplot, the horizontal line inside the box indicates the average and the height of the box indicates the interquartile range including 50% of the overall points.

Variations in population and grain output show significant differences among four types of grain insecurity (Figure 3). The population increased, on average, by 342,042 people in cities of grain insecurity, which was approximately 9 times the grain security. The grain output decreased, on average, by 90,438.6 tons. Furthermore, the variations in total population and grain output have significant differences between groups. Approximately 31.4% and 33.2% of the cities identified as having grain insecurity experienced a population decrease and grain output increase, respectively. On the demand side, the decreased surpluses and increased deficits were associated with a larger population increase than the other types. For the decreased deficits, there were approximately 54.7% cities that had a decreased population. On the supply side, all cities with decreased deficits experienced an average grain output increase of 123,475.1 tons. For the decreased surplus and increased deficit, approximately 19.4% and 20.8% of the cities presented with increased grain output, respectively. 

**Figure 4:**
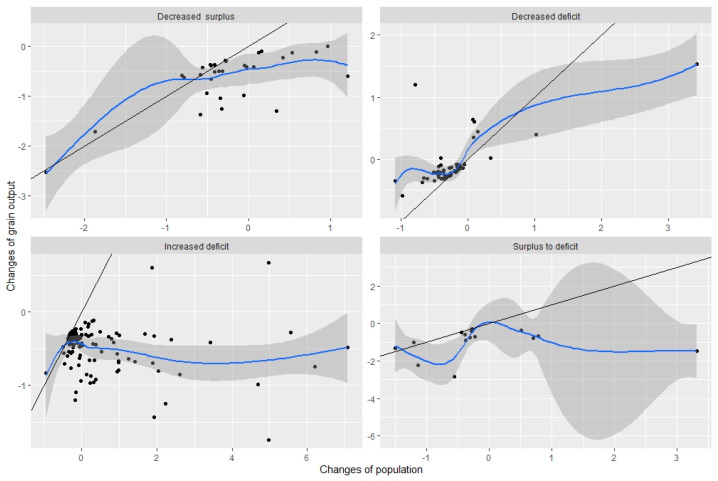
Normalized variations in the population and the grain output for four types of grain insecurity. The horizontal axis indicates the normalized variation of the population, and the vertical axis indicates the grain output. A positive value indicates that the change is above the mean and a negative value indicates that it is below the mean; the black points denote 220 cities in China. The solid black line is the linear equation (y = x). If points are above the 1:1 fit line, the changes in the grain output are more significant than those in the population, and vice versa.

Population and grain production have different effects on the regional grain system (Figure 4). Approximately 67.3% of the cities identified as having grain insecurity fall into the third quadrant, which is of negative value both for population and grain production. This indicates that the variations in the population and the grain production in these cities are below the mean. Specifically, approximately 71% of the points in decreased surplus, 83.3% of the points in decreased deficit, 58.3% of the points in increased deficit, and 73.3% of the points in surplus to deficit fall into the third quadrant, respectively. There were another 27.7% of cities identified as having grain insecurity in the fourth quadrant. This indicates that the variations of the population are above the mean, while the variations of the grain output are below the mean. Thus, approximately 29% of the points in decreased surplus, 40% of the points in increased grain deficit, and 26.7% of the points in surplus to deficit fall into the fourth quadrant, respectively. For the decreased deficit, there were seven points in the first quadrant, indicating different variations in the grain production. These cities also had larger variations in the population and grain output than the mean of the total values.

Most cities identified as having decreased surplus, increased deficit, or gone from surplus to deficit experienced a larger population change than grain production. This shows that very few points in these cities were below the linear equation (y = x), indicating that the population had a larger share than the grain production to explain the grain insecurity on a regional scale. On the contrary, for the decreased deficit, almost all points lie above the linear equation. This indicates that the variations in grain production were more obvious than those in the population. 

### 3.3. Uneven Distribution of Cultivated Land under Four Types of Grain Insecurity

**Figure 5:**
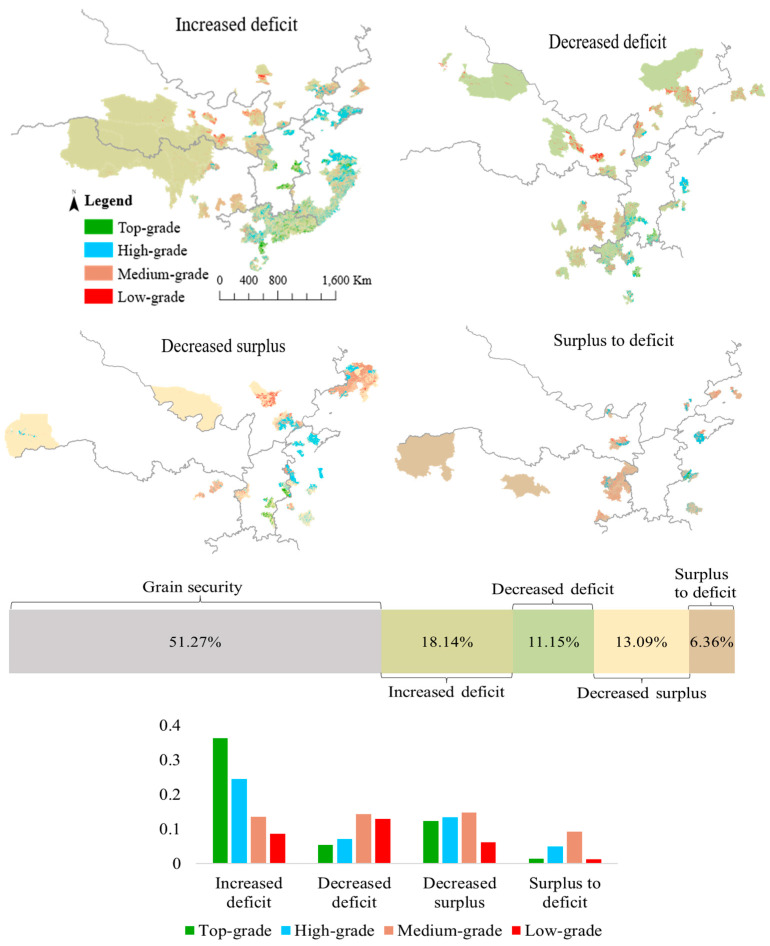
Spatial distribution of cultivated land varying with four types of grain insecurity. Where the colored bar below denotes the total proportion of cultivated land, the right bar chart denotes the proportion of cultivated land with top-grade, high-grade, medium-grade, and low-grade.

The grain-insecure cities occupied approximately 48.73% of the cultivated land (Figure 5). The increased deficit occurred on the largest cultivated land area (18.14%). This was followed by the decrease in grain surplus, which occurred on approximately 13.09% of cultivated land. The decrease in deficit occupies approximately 11.15% of the cultivated land. Only 6.36% of cultivated land was located in cities identified as having gone from surplus to deficit. 

However, there were stark differences in the quality of cultivated land between the four types of grain insecurity. There was a total of 55.4% of top-grade land, 49.8% of high-grade land, 51.9% of medium-grade land, and 28.9% of low-grade land distributed in cities with grain insecurity. The medium-grade land was predominant in the areas of decreased deficit, decreased surplus, and surplus to deficit, but not in areas of increased deficit. This shows that the increased deficit occurred in approximately 24.37% of high-grade land, which was more than in medium-grade land (13.61%). The top-grade land had a small scale, but 36.2% was distributed in cities with an increased deficit. The decreased surplus occurred in approximately 12.3% of the top-grade land and 13.32% of the high-grade land. Approximately 8.61% and 6.17% of low-grade land was in increased deficit and decreased surplus, respectively. Moreover, this shows that the spatial distribution of high-quality cultivated land was a long distance from the low-grade land. The barycenter of the top-grade land is located in the south of the producing areas. The low-grade land was mainly distributed in the north of the producing areas along the northeast to the southwest of China.

## 4. Discussion

### 4.1. Contributions and Uncertainties

This study characterizes grain insecurity on the regional scale as four types by comparing the variations in grain supply–demand conditions during the past 10 years, which is different from previous studies. One of the main findings here is that, from 2008 to 2018, approximately 61.6% of prefecture-level cities in China were in an unsustainable grain-producing system, while the proportion was only 48% in 2012, according to our previous findings [16]. The evidence of such unsustainable and deteriorating conditions of regional grain-producing systems has been presented in other studies. For example, the gap between grain supply and demand is increasing, and grain shortage has become a common phenomenon in 21 cities in Guangdong Province [17]. Even in major producing areas, the proportion of non-grain production is shown to have an increasing trend [18]. These cities with grain insecurity should be identified early on. Another related finding is the enlarged regional disparities. These show that northern and central China experienced more sustainable grain-producing conditions compared to the southern and southwestern regions, which is consistent with previous findings that the grain supply pattern has changed from the south to the north in China [7,15]. 

In this study, the determinants of the evolution of the regional grain system are further uncovered. Our findings support grain output as decisive for sustainable grain supply–demand conditions on the city scale. This shows that the majority of cities with grain insecurity experienced a grain output decrease and a population increase. In contrast, almost 90% of cities presenting with grain security experienced a significant increase in grain output. The evolution of the regional grain-producing system generally keeps in step with the variations in grain output. We thus infer that there is mainly a supply-side crisis for these unsustainable grain systems. In addition, there were a few outliers on the far top side of population and bottom side of grain output, as presented in Figure 3. These outliers are characterized as mega-cities, including Beijing, Shanghai, Guangzhou, Shenzhen, and Chongqing.

Another finding is the disharmony between grain insecurity and the distribution of cultivated land. This shows that cities with an unsustainable grain-producing system occupy large tracts of cultivated land, especially high-quality land. Thus, high-quality cultivated land is, to some extent, in the wrong place. Yet it reveals that grain security in China is more dependent on the amount of cultivated land than on soil nutrient levels to sustain the primary grain supply. As in the FAO report (2008) [4], nearly 80% of increased grain production in developing countries is based on cropping intensity. In addition, cities with an increase in deficit should be emphasized, for they especially cover large tracts of high-grade and top-grade cultivated land.

This study provides shreds of evidence for a long-lasting debate on what city size is beneficial for grain security. A common belief is that large cities destroy grain supply conditions by encroaching on cultivated land in urban–rural fringe areas [19,20]. The opposite view supports the megacities strategy in the sense that migration of the population to megacities is beneficial to protecting cultivated land in small cities [21]. In this study, we found that most megacities present grain insecurity due to a decrease in grain output and the dense population. Combined with the fact that megacities in China cover large tracts of high-quality cultivated land, we advocate for small cities rather than megacities in the urbanization and policy-making processes to ensure grain security. In general, the development of megacities would indirectly result in the loss of grain productivity. 

There were some uncertainties and limitations in this study. On the supply side, some intermediate and comprehensive factors were not included here. For example, grain supply is substantially influenced by grain reserves, trade, agricultural management, and the like [22]. However, it is simply proxied by grain output in this study, which provides limited insight into grain insecurity patterns. On the side of grain demand, we regard the per capita share of grain as a constant in different periods and regions, which neglects the variability of dietary habits and would further reduce the heterogeneity of grain demand at both temporal and spatial scales. We suggest consideration of these uncertainties and limitations in the future.

### 4.2. Enlarged Disparities among Producing, Consuming, and Balanced Areas

**Figure 6:**
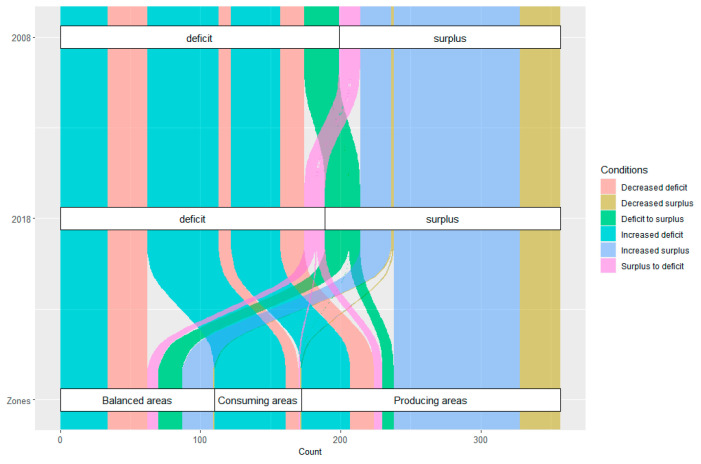
The proportion of cities with different types of grain insecurity in the producing, consuming, and balanced areas. The vertical axis indicates the period from 2008 to 2018 and the horizontal axis indicates the number of cities.

In China, the high level of holistic grain security is based on a series of initiatives issued by the government, including the common but differentiated responsibilities of grain production, the practice of holding provincial governors responsible for the ‘rice bag’, etc. A top-down strategy regarding the designation of producing, consuming, and balanced areas at the provincial levels is the basis for and crucial to the pooling of the responsibilities of national grain security [23]. Supplementary evidence is presented here to compare the disparities in grain supply–demand conditions between producing, consuming, and balanced areas (Figure 6). 

The producing areas have seen a more sustainable grain system during the last decade. Approximately 48.67% of cities had an increased grain surplus, which mainly benefits from the steady increase per unit yield and a generous amount of cultivated land. Two hotspots of high–high associations were identified in the producing areas, indicating the improvement of grain supply-demand conditions during the period. However, quite a few cities in the producing areas were experiencing grain insecurity, mainly along the eastern coast and in western China. Such findings reveal the increased imbalance of the regional grain system in the producing areas. 

Variations in grain supply–demand conditions in the consuming and balanced areas are unsatisfactory. More than 85% of the consuming regions continue to experience grain insecurity. The increased grain deficit accounts for approximately 82%. In the balanced areas, more than 60% of the cities saw a decrease in grain deficit. More than 71% of cities were in grain deficit in 2008, while the proportion decreased to 63% in 2018. Additionally, approximately 15% of the cities experienced a change from deficit to surplus. Accordingly, we infer the degradation of grain systems in consuming and balanced areas.

In addition, there are larger regional disparities in the producing, consuming, and balanced areas, as well. It is certain that the producing areas have sustainable conditions for grain supply and demand and play a crucial role in feeding the domestic population. According to statistical data, the producing areas provide an average of 76% of the total grain output and directly feed more than half of the population. Meanwhile, the consuming areas deliver approximately 5% of the total grain production to feed 20% of the total population. And, the balanced areas provide almost 19% of grain production and nourish nearly 22% of the population. In this regard, quite a few consuming and balanced areas could not satisfy basic grain demand. Furthermore, the consuming and balanced areas persisted in a significant decrease in grain output in the past decade. It appears that the increase in grain production in the producing areas could compensate for the reduction in the consuming areas and thereby sustain a holistic grain balance. The transregional grain circulation is therefore necessary to close the gap, especially for the consuming and balanced areas. 

The strategy of intensive farming and differentiated responsibilities for grain security is appreciated, but simultaneously leaves a legacy for the whole grain-producing system. Regional grain security is challenged by some supply-side problems. For one, most cities with grain security have long benefited from the increase in grain yield per unit but are trapped in the marginal utility of cultivated land. It is noted that grain yield decreased from 70 kg in the 1960s to less than 20 kg in 2012 when 1 kg of chemical fertilizer was injected into the cultivated land [24]. There is limited potential to increase the yield per unit even with the help of technological advances and investment [25]. One reason for this is that the phenomenon of non-grain production on cultivated land is prevalent in China, triggering the reduction in grain supply and the degradation of cultivated land [26]. Farmers in poverty-stricken regions are motivated to seek high profits by converting cultivated land from grain planting to other agricultural production [27]. Last but not least, changes in grain production patterns aggravate the uneven distribution of natural resources, especially the mismatch of agricultural water. This complicates the transregional grain chain and increases the transport distance, which implies a higher cost for the dense populations in large cities when access to available food is required.

### 4.3. Sustainable Approaches to Regional Grain Security

Informed by the legacy from the grain supply side, we consider that enlarged regional disparities and the strategy of differentiated responsibilities regarding grain output should be noted. Still, they should be based on the premise of a degree of self-sufficiency. Undoubtedly, cities with grain security assume the primary responsibility for domestic grain production. They are advised to seek the ceiling and floor of grain supply. However, there remains tension between investing in agricultural production and eliminating poverty. Therefore, the priority for those cities trapped in poverty is to balance agricultural production and economic development. An effective approach is to establish the grain supply ceiling accompanied by related initiatives, including ecological compensation and transfer payment for the protection of cultivated land. The other cities in the producing areas should assume extended responsibilities for grain production to meet the increased demand from the balanced and consuming areas, which relates to the national grain security baseline. In this vein, more attention should be paid to preventing non-grain utilization as well as protecting high-quality cultivated land. 

We cannot rely exclusively on cities in the main producing areas to provide sufficient food for the nation. Environmental sustainability should be embedded in the management of the regional grain system. For the cities in consuming areas, the priority is to share part of the responsibilities for grain security under the premise of economic prosperity. There is very high-quality cultivated land in the consuming areas. It is suggested that these cities establish the baseline for grain self-sufficiency, which means limited and specific responsibilities for grain output. Furthermore, the cities in consuming areas require optimal utilization of natural resources based on understanding their interaction. For example, the planting of soybeans is appropriate in southern China, in the sense that natural resources involving solar radiation and water provisioning in the south are superior to those in the north. Grain security, in many contexts, underscores sustainable grain production and the long-term resilience of agricultural practices that increase productivity and adaptability. A novel paradigm of the water–energy–food nexus can potentially assist in coupling sustainable grain systems with the utilization of resources [28,29]. 

In general, regional grain security is central, but has not been widely brought to the forefront due to the long balance of grain supply–demand conditions on the national scale. We propose that intensive management and differentiated responsibilities are indispensable for national grain security. However, the legacy left to the whole grain-producing system should be mentioned. As we suggested above, interregional cooperation for grain transportation should be based on local self-sufficiency.

## 5. Conclusions

There is a great share of cities that more or less present with grain insecurity, which should be prioritized early on. This shows that 220 out of 357 cities are in grain insecurity in the mainland of China. The largest proportion of cities are in an increased deficit and 15 cities have experienced absolute changes from surplus to deficit. The enlarged disparities occur on the regional scale. Almost all cities in the south of China present grain insecurity, which is predominated by an increase in the grain deficit. Most cities in the southwest are in increased grain deficit and experience changes from surplus to deficit.

Cities with unsustainable grain-producing systems are subject to the dual effects of both increased population and decreased grain production. This shows that the population in grain-insecure cities increase, on average, by 342,042 persons, which is approximately 9 times that of the grain-secure cities. The grain production decreases, on average, by 90,438.6 tons. Approximately 67.3% of grain-insecure cities fall into the third quadrant, indicating the variations in population and grain production below the mean. However, most of these cities are above the linear equation (y = x). We conclude that grain production is substantially responsible for grain insecurity compared to the variations in the population.

Grain-insecure cities occupy a large share of high-quality cultivated land, indicating the mismatch of grain productivity and supply–demand conditions, which is of great importance for regional grain security. There is approximately 55.4% of top-grade land, 49.8% of high-grade land, 51.9% of medium-grade land, and 28.9% of low-grade land in grain-insecure cities. It is noted that the increased deficit and decreased surplus, indicating a severe state of the regional grain-producing system, occurs in 48.5% of top-grade land, 37.7% of high-grade land, and only approximately 14.7% of low-grade land.

## Data Availability

The statistical data are available from the corresponding author upon reasonable request.
